# Human-based dynamics of mental workload in complicated systems

**DOI:** 10.17179/excli2019-1372

**Published:** 2019-07-11

**Authors:** Mohammad-Javad Jafari, Farid Zaeri, Amir H. Jafari, Amir T. Payandeh Najafabadi, Narmin Hassanzadeh-Rangi

**Affiliations:** 1 Department of Occupational Health and Safety Engineering, School of Public Health and Safety, Shahid Beheshti University of Medical Sciences, Tehran, Iran; 2 Proteomics Research Center and Department of Biostatistics, Faculty of Paramedical Sciences, Shahid Beheshti University of Medical Sciences, Tehran, Iran; 3 Medical Physics & Biomedical Engineering Department, School of Medicine, Tehran University of Medical Sciences, Tehran, Iran; 4 Department of Actuarial Science, Faculty of Mathematical Sciences, Shahid Beheshti University, G.C. Evin, 1983963113

**Keywords:** mental workload, ergonomics, archetype, review, system dynamics

## Abstract

As a dynamic system in which different factors affect human performance via dynamic interactions, mental workload needs a dynamic measure to monitor its factors and evidence in a complicated system, an approach that is lacking in the literature. The present study introduces a system dynamics-based model for designing feedback mechanisms related to the mental workload through literature review and content analysis of the previous studies. A human-based archetype of mental workload was detected from the data collection process. The archetype is presented at various stages, including dynamic theory, behavior over time, leverage points and model verification. The real validation of the dynamic model was confirmed in an urban train simulator. The dynamic model can be used to analyze the long-term behavior of the mental workload. Decision-makers can benefit from the developed archetypes in evaluating the dynamic impact of their decisions on accident prevention in the complicated systems.

## Introduction

The reason for errors, crashes, accidents and disasters made by human can be due to unbalance mental workload resulting in overload and underload situations exposing operators to approach or exceed the redlines of their performance (Xie and Salvendy, 2000[36]; Paxion et al., 2014[27]; Young et al., 2015[39]; Wascher et al., 2016[33]). On the other hand, the balance in the workload reduces the human error and increases the task performance of operators (Xie and Salvendy, 2000[36]; Yu et al., 2016[40]; Zhao et al., 2016[41]). Therefore, the concept of mental workload and mechanism of its effect on task performance in different human-machine systems is considered by practitioners and researchers in a variety of cognitive activities, such as conventional driving (Allahyari et al., 2014[1]; Hassanzadeh-Rangi et al., 2014[18]; Yan et al., 2019[37]), automated driving (Ko and Ji, 2018[22]), train driving (Balfe et al., 2017[6]), nuclear power plants (Choi et al., 2018[12]), advanced surgery programs (Cavuoto et al., 2017[9]), air traffic monitoring (Dasari et al., 2017[14]), control rooms (Melo et al., 2017[24]), workplace activities (Chen et al., 2017[11]), information technologies (Buettner, 2017[7]) and other complex human-machine systems (Xiao et al., 2015[35]). Few conceptual frameworks are available for understanding mental workload mechanism based on the static relationship extracted from traditional statistics (Xie and Salvendy, 2000[36]). However, the mental workload and its evidence, i.e. fatigue and other psycho-physiological responses, are dynamic phenomena affected by the various factors in different time intervals (Charbonnier et al., 2016[10]). System thinking can be a successful approach in examining the dynamic pattern of change and drawing behavior over time instead of static snapshots (Senge, 2006[30]). Systems thinking can be conducted by system dynamics approach. Causal-loop archetypes in the form of stock-flow diagrams describe the structure of a system in system dynamics. A stock-flow diagram consists of state (level), flow (rate) and auxiliary (constant) variables. Math equations determine the relationships between variables in stock-flow diagrams. System dynamics is supported by the graphics simulation programs including Vensim, Powersim and i-think to describe behavior over time (Sterman, 2001[31]; Yim et al., 2004[38]; Azar, 2012[4]). There are no applications of system thinking and system dynamics in mental workload research. This work, therefore, tries to fill these gaps by developing the mental workload archetypes and their behavior overtime with a focus on task demand and human performance.

## Methods

### Literature search

Contributing factors and their relations as well as conceptual models were extracted from the literature review. We reviewed three databases including Scopus, Web of Science and Pubmed International Profile for related articles published between 2007 and 2018 and related abstracts of international conferences held between 2014 and 2018. The keywords were (workload OR mental workload) AND (model OR measurement OR evaluation OR assessment OR predicting OR survey OR rating OR scale OR Index OR questionnaire). 

We screened 339 articles out of 979 articles based on the inclusion criteria, including original article, full-text article, publication in English, quantitative data analysis, transparency in presenting research methods and results, and other quality rating appraisal (Khosravi et al., 2014[19]; Mohammadi et al., 2018[26]).

### Content analysis 

We extracted the contributing factors of mental workload and their relations through reviewing the abstracts and related parts of 358 included articles. Also, we reviewed, if necessary, the full text of the included articles for further certainty. We extracted a static conceptual model and its assumptions by reviewing the full text of 87 articles. We simultaneously categorized the reviewed studies on the basis of reference type, study design, data collection tools, field, setting, analytical method, variables, and key results (see Supplementary Table 1). Direct content analysis followed by theme analysis helped to extract contributing factors, evidence, and conceptual static and dynamic models (Khosravi et al., 2013[20], 2014[21]; Asilian-Mahabadi et al., 2018[3]; Mohammadi et al., 2018[25]). Peer review confirmed that the data saturation has occurred in the content analysis, and themes are properly extracted.

### System dynamics modeling

At first, we explained the real problem in the context of mental workload monitoring. Then, we extracted the contributing factors of mental workload in the framework of a static model. 

In the second stage, we determined the dynamic hypothesis between the variables of the static model based on the statistical relationships of previous studies (Mohammadi et al., 2018[25]). The Vensim software (version 5.10) was used to plot the mental workload archetype. In this software, the archetype variables including stocks, flows and, auxiliaries, were displayed in rectangles, hourglass shapes and plain texts, respectively. In this software, the stock variables displayed in rectangles present the level of the archetype variables over time. The flow variables displayed in hourglass shapes exhibit the rate of stock variables over time. The auxiliary variables displayed in the plain texts (surrounded by the brackets in some cases) indicate the constant values and other variables of the archetype. One-way arrows show the cause-effect relationship between two variables. Circular arrows tagged with A and B show the balancing feedbacks and reinforcing feedbacks, respectively (García, 2019[17]). 

The third stage was the definition of the stock-flow diagrams to the math equations ruling on the variables. The formulation in the Vensim software was done through simple math equations such as integration. The look-up function in the Vensim software allowed us to estimate the internal relations of some variables in relation to each other over time. 

The fourth stage was the simulation and testing process. We extracted the simulation outputs as the graphs displaying the relationships between the variables over time. There are different methods of validation, including the structure test, boundary test, dimension consistency, parameter verification, extreme conditions, and structurally oriented behavior test to validate the stock-flow diagrams (Yim et al., 2004[38]; Babader et al., 2016[5]). The structure assessment and the Vensim features were used to perform dimension checks, extreme conditions analysis, sensitivity test, and reality check. Sterman (2001[31]) and García (2019[17]) provide detailed characteristics of system dynamics.

The real validation of the dynamic model was carried out in an urban train simulator. Twelve healthy male metro train drivers (age range of 25 to 45 years; driving experience of 1 to 5 years; physically and mentally healthy; drug free; and nonsmokers) volunteered to participate in an experimental study. The experimental route was a part of Tehran metro line 1 (39 km, 20 min). The scenarios were designed according to the real train operation and the rule books. The experimental tasks were divided into two categories: getting ready to drive and driving between stations. The getting ready to drive consisted of changing the train's direction; completing the train dispatching procedure, including train activation, train safety activation, and check-up; observing and obeying signaling indications and train warning systems. The driving between stations consisted of driving the train along the track; obeying speed limits and other train protection orders; monitoring the surrounding environment; observing and obeying signaling indications and train warning systems; stopping the train at a specific area in the stations; opening and closing train doors; entering and leaving stations within the speed limit. A high-fidelity simulator was used for the experimental driving tasks in this study. Train drivers evaluated their workload status by using the integrated workload scale (IWS) during the particular driving tasks. The mental workload can be assessed by specific subjective scales. The IWS is a self-rating tool to assess mental workload in real-world settings, and it is sensitive to several environmental and task-related factors in railway industry (Pickup et al., 2005[28]; Wilms and Zeilstra 2013[34]). In these methods, operators are able to rate work demands themselves on a numerical or graphical scale (Young et al., 2015[39]). 

The environmental parameters in the simulated cabin were maintained to the same conditions of the real cabin. The illumination level (in Lux), the wet bulb globe temperature (in °C) and the noise level (in dBA) were measured to ensure that these parameters were consistent between the real and simulated environment. The average judgment of drivers from their mental workload in different situations was entered manually in the reference mode of the Vensim software. 

**Figure 1:**
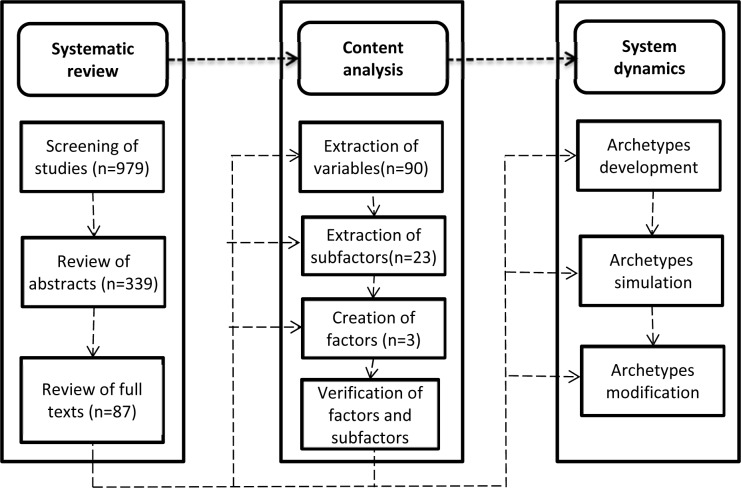
Flow diagram of the study design

In the fifth stage, the model behavior over time was utilized to design the leverage points. The leverage points could have advantages for decision makers to improve the complicated systems (Mohammadi et al., 2018[25]). The flow chart of the study design is presented in Figure 1(Fig. 1). 

## Results and Discussion

### Conceptual model and real problem

According to previous reports and content analysis, we extracted the main variables (or measures) probably useful for the assessment of the mental workload in a complex system. Accordingly, the result was 90 measures (variables) in this regard. These variables were further condensed according to some of the common themes that were later classified under 9 categories. These variables were grouped into 23 themes (sub-factors) and three factors (or evidence), namely 1) task demand and job characteristics 2), external and environmental stress, and 3) individual capabilities and characteristics. (Supplementary Table 2 shows summarized factors and sub-factors, along with the strength of their evidence.)

Previous research on the mental workload underlined three main gaps, which may prevent previous findings widely used in practical fields in the complex systems. First, the studies are limited to one or two variables. Second, the results are too narrow with no approaches for generalizing the results. Third, all results are static, where just one variable has been assigned to mental workload during the study. In contrast, mental workload is usually a time-based issue, and the type of demand assigned to operators is a dynamic pattern.

To address these gaps, the human-based archetypes were developed by system dynamics to account for the various factors of mental workload. Using this dynamic model, the dynamic relations among mental workload and related contributing factors were depicted to optimize mental workload balance resulting in improved performance. The human-based archetype of mental workload is presented as follows.

### Human-based archetype of mental workload

#### Dynamic theory

The basic principles of the physiological dimensions of mental workload follow the assumption that physiological responses depend on physiologically active mechanisms in high-demand jobs (Ryu and Myung, 2005[29]).

The alterations in resource capacity and operator performance can be seen in the physiological feedbacks (Mehler et al., 2009[23]). Moreover, the mental workload of operators can be monitored within the operation phase using physiological measures (Durkee et al., 2013[16]). Such measures are passive with no need for an overt response from the operator and allow continuously monitoring the mental workload (Cain, 2007[8]). The physiological workload measures are hardly widely used in the practical fields because of the need for specialists for application and analysis (Teo et al., 2015[32]; Chuang et al., 2016[13]). 

**Figure 2:**
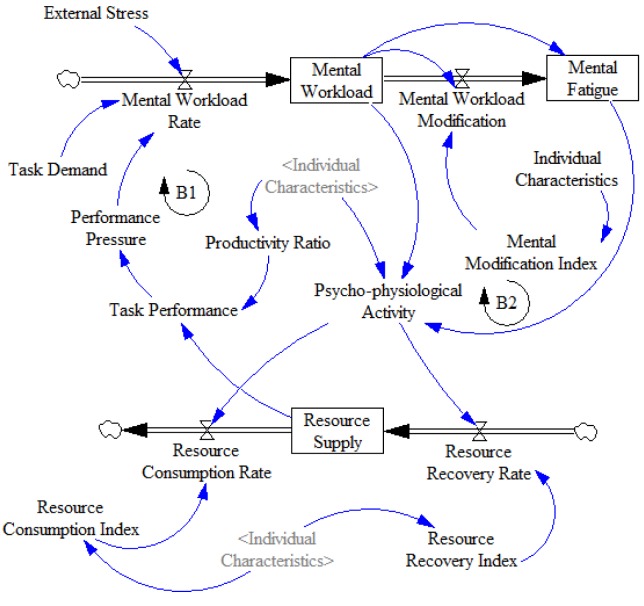
The human-based archetype of mental workload

Figure 2(Fig. 2) shows the archetype of resource supply consisting of two balancing loops (B1 _Mental activation_, and B2 _Mental burnout_). According to the theory of limited resources, the human mind has limited resources for thoughtful processing (Alvanchi et al., 2011[2]). As shown in B1 _Mental activation _loop, the level of resource supply is the result of the interaction between resource recovery rate and resource consumption rate during the operating period. Thus, a resource consumption rate higher than resource recovery rate leads to resource limitation. The resource limitation decreases task performance and consequently increases performance pressure and mental workload. As a result, in a short time, brain and cardiovascular activities increase the number of available resources. As shown in B2 _Mental burnout_, in a long time, the excessive consumption of resources leads to decreased task performance. In return, the decreased task performance increases the performance pressure, mental workload and mental fatigue, respectively (Alvanchi et al., 2011[2]; Young et al., 2015[39]).

Individual characteristic is a key auxiliary variable that influences the internal variables, including psycho-physiological response, workload modification, resource consumption, resource recovery, and productivity ratio and ultimately the recognized feedback loops. (The formulated model and its equations are presented in Supplementary Table 3.)

**Figure 3:**
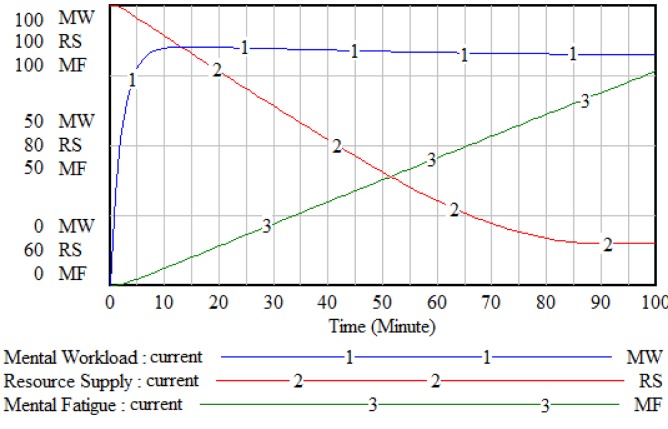
The behavior pattern of resource supply and mental workload archetype over time

The typical behavior of the resource supply and mental workload interaction over time is shown in Figure 3(Fig. 3). At the beginning of a complex operation, as mental workload increases, the B1 _Mental activation _loop will be activated and the resources will be available. Simultaneously, the new resources are replaced by resource recovery process (Alvanchi et al., 2011[2]). Although the level of workload is under control until the end of the operation, the cumulative fatigue activates the B2 _Mental burnout_ loop. As a result, the available resources gradually decrease to 50 % of its initial level. The behavioral pattern of mental workload and resource supply in this study is consistent with the conceptual patterns drawn in the previous studies (De Waard, 1996[15]; Young et al., 2015[39]).

#### Leverage points

The leverage point of resource supply and mental workload archetype relies on the balancing B2 _Mental burnout_ loop. This archetype focuses more on the human aspect of complex systems. Behavior over time of this archetype shows that the mental overload leads to overcoming the resources consumption rate to resources recovery rate. As a result, mental fatigue appears in the short-term and mental burnout in the long-term. Therefore, moderation of mental demand and external stress should be proportional to individual characteristics. This moderation, on the one hand, should be made in such a way as to provide an opportunity to recover resources over time. On the other hand, the performance pressure should be appropriate as the other input of the mental workload.

### Verification of the dynamic model

The verification methods used in this study were the structure assessment, the extreme conditions, the sensitivity analysis, and the real validation. The structure assessment tests verify that the model is compatible with real-world knowledge (Sterman, 2001[31]). In this study, the content analysis is used to develop the static and dynamic models of mental workload. Since the content analysis is based on the previous studies and the data saturation, the structure assessment has been done during the developing process of the conceptual models. As well as, a significant agreement was found between two reviewers in the thematic coding of the contents in accordance with the results of the inter-rater reliability (kappa=0.91). Furthermore, other structure tests were performed through peer review and expert verification. The blinded experts, who participated in four interviews and a focus group, logically approved the stock-flow diagrams. In addition, these experts reported that the real operation confirmed the results of behavior over time extracted from the Vensim software for mental workload modeling. 

**Figure 4:**
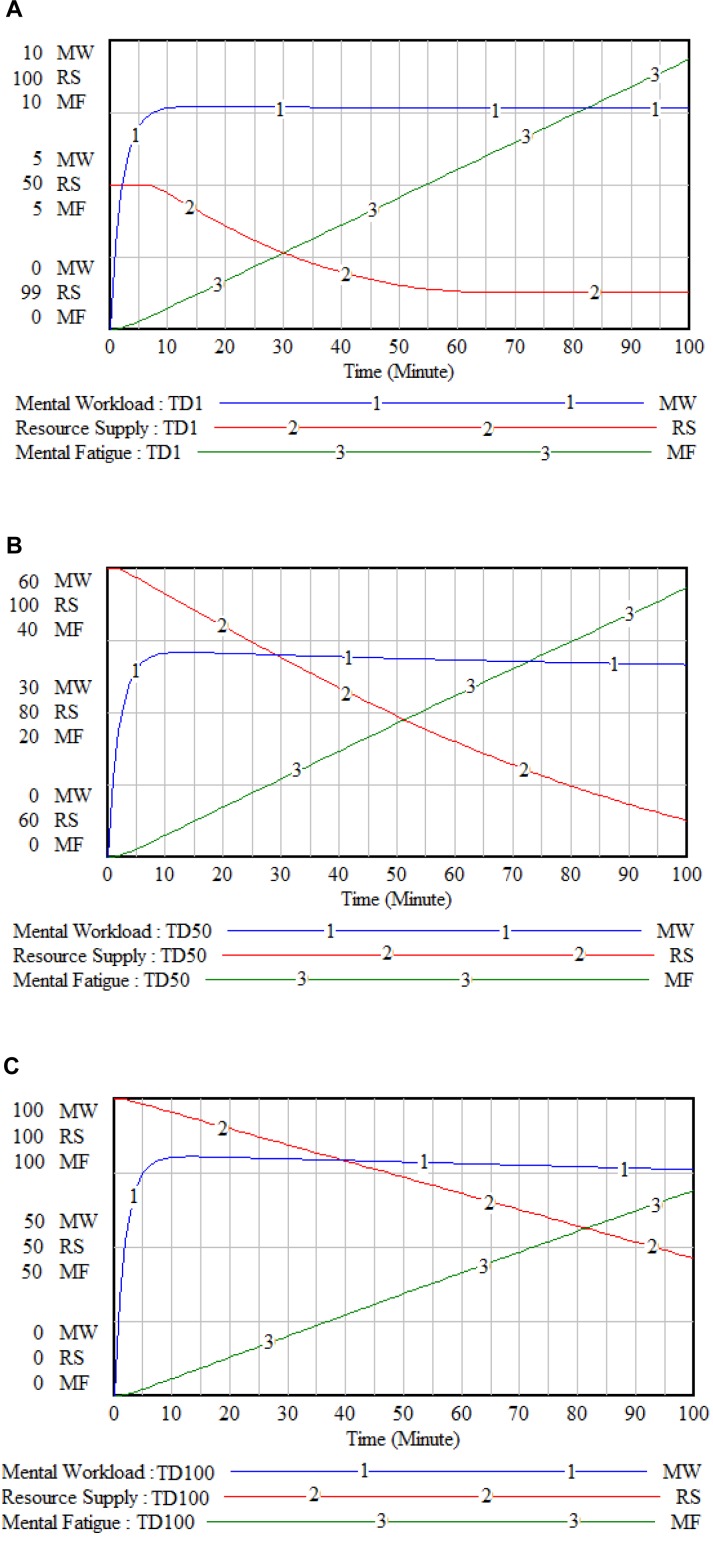
Extreme conditions (A: 1 % task demand, and C: 100 % task demand) and sensitivity analysis (A: 1 % task demand, B: 50 % task demand and C: 100 % task demand) of the dynamic model

The results of behavior over time for the human-based archetype of mental workload showed the behavior of the dynamic model is logical at different levels of the task demand. Sensitivity analysis (Figure 4A, B and C(Fig. 4)) shows that as long as the task demand increases, the mental workload increases, and the resource supply decrease. The extreme conditions analysis (Figure 4A and C(Fig. 4)) showed at the lowest level of duty demand, the trend of increasing mental workload is minimal and related to the performance pressure. In these conditions, the trend of increasing mental fatigue and reducing resource supply is minimal. At the highest level of task demand, the trends of increasing mental workload and fatigue, and reducing resource supply are maximal. (Supplementary Figure 1 shows the Vensim software runs under different conditions on various variables in the dynamic model.) 

**Figure 5:**
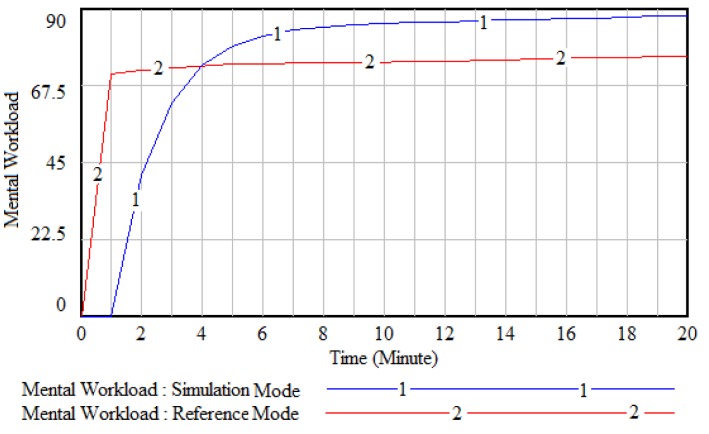
Real validation of the human-based model

Comparison of the reference mode and the model simulation (Figure 5(Fig. 5)) indicates that the dynamic model can estimate the mental workload close to reality in the urban train simulator. 

The reference mode displays the actual pattern of mental workload over time during the experimental driving tasks. The simulation mode displays the simulated pattern of mental workload over time according to the experimental driving data. Figure 5(Fig. 5) shows that at the beginning of the experimental tasks (getting ready to drive), as long as the task demand increases, the mental workload increases in both the reference mode and the simulation mode. The behavioral pattern of mental workload is under control and similar during driving between stations until the end of the operation in both reference mode and simulation mode. 

## Overall Discussion and Conclusion

### Contribution to the complicated system

In the present study, a simulation approach was used to understand the complexities of mental workload in complex systems. Human performance management encounters numerous challenges in complex operations due to the dynamic nature of complex operations and interactions between the factors affecting the mental workload. In this study, the dynamical mechanism of mental workload and its interaction with task demand and resources supply variables were simulated. The simulated models in this study can be applied to 1) identify changes in mental workload and its outcomes, such as task performance and mental fatigue during operations. 2) extract corrective and preventive measures based on a dynamic approach to optimize workload during complex operations. The current study in line with the previous studies has shown that both overload and underload could have adverse effects on humans and their performance (Young et al., 2015[39]).

### Limitations and future research

In this study, factors affecting the workload were reviewed over a ten-year period. Although data saturation was achieved in the extraction of themes and factors, the researchers encountered a lack of studies to confirm some of the relationships between variables. To overcome this shortcoming, content validity and expert judgment were used (Hassanzadeh-Rangi et al., 2014[18]). An updated review can help improve the results of this study in the future. In this study, various validation methods were used to confirm the conceptual model, archetypes and behavior over time. For more validity and generalizability of the dynamic model, it is necessary to study the current archetypes in future empirical studies.

## Conclusion

In the present study, the conceptual framework of mental workload and its contributing factors was developed using literature review. The system dynamic approach indicated the interactions between the factors affecting the mental workload in the form of behavioral patterns (archetypes). The behavior over time of mental workload archetype was simulated with a focus on the human-based variables. The result showed that the dynamic models could be used to analyze the long-term behavior of the mental workload, taking into account the various contributing factors and the associated uncertainty. In addition, this study can be considered as a starting point for future research through a combination of mental workload and system dynamics.

## Acknowledgement

This paper was submitted as part of a thesis for the Ph.D. degree in Occupational Hygiene, School of Health and Safety, Shahid Beheshti University of Medical Sciences, Tehran, Iran. This work was supported by Shahid Beheshti University of Medical Sciences (Grant number: 10692). 

## Conflict of interest

None to report. 

## Supplementary Material

Supplementary material

## References

[1] Allahyari T, Rangi NH, Khalkhali H, Khosravi Y (2014). Occupational cognitive failures and safety performance in the workplace. Int J Occup Saf Ergon.

[2] Alvanchi A, Lee S, AbouRizk S (2011). Dynamics of working hours in construction. J Construct Eng Manag.

[3] Asilian-Mahabadi H, Khosravi Y, Hassanzadeh-Rangi N, Hajizadeh E, Behzadan AH (2018). A qualitative investigation of factors influencing unsafe work behaviors on construction projects. Work.

[4] Azar AT (2012). System dynamics as a useful technique for complex systems. Int J Indust Syst Eng.

[5] Babader A, Ren J, Jones KO, Wang J (2016). A system dynamics approach for enhancing social behaviours regarding the reuse of packaging. Exp Syst Applic.

[6] Balfe N, Crowley K, Smith B, Longo L, Longo L, Leva MC (2017). Estimation of train driver workload: extracting taskload measures from on-train-data-recorders. Human mental workload: models and applications.

[7] Buettner R (2017). The relationship between visual website complexity and a user’s mental workload: A NeuroIS perspective. Inform Syst Neurosci.

[8] Cain B (2007). A review of the mental workload literature.

[9] Cavuoto LA, Hussein AA, Vasan V, Ahmed Y, Durrani A, Khan S (2017). Improving teamwork: evaluating workload of surgical team during robot-assisted surgery. Urology.

[10] Charbonnier S, Roy RN, Bonnet S, Campagne A (2016). EEG index for control operators’ mental fatigue monitoring using interactions between brain regions. Exp Syst Applic.

[11] Chen J, Taylor JE, Comu S (2017). Assessing task mental workload in construction projects: a novel electroencephalography approach. J Construct Eng Managem.

[12] Choi MK, Lee SM, Ha JS, Seong PH (2018). Development of an EEG-based workload measurement method in nuclear power plants. Ann Nucl Energy.

[13] Chuang CY, Lin CJ, Shiang WJ, Hsieh TL, Liou JL (2016). Development of an objective mental workload assessment tool based on Rasmussen's skill-rule-knowledge framework. J Nucl Sci Technol.

[14] Dasari D, Shou G, Ding L (2017). ICA-derived EEG correlates to mental fatigue, effort, and workload in a realistically simulated air traffic control task. Front Neurosci.

[15] De Waard D (1996). The measurement of drivers' mental workload.

[16] Durkee K, Geyer A, Pappada S, Ortiz A, Galster S, Schmorrow DD, Fidopiastis CM (2013). Real-time workload assessment as a foundation for human performance augmentation. Foundations of augmented cognition.

[17] García JM (2019). Theory and practical exercises of system dynamics.

[18] Hassanzadeh-Rangi N, Farshad AA, Khosravi Y, Zare G, Mirkazemi R (2014). Occupational cognitive failure and its relationship with unsafe behaviors and accidents. Int J Occup Saf Ergon.

[19] Khosravi Y, Asilian-Mahabadi H, Hajizadeh E, Hassanzadeh-Rangi N, Bastani H, Behzadan AH (2014). Factors influencing unsafe behaviors and accidents on construction sites: a review. Int J Occup Saf Ergon.

[20] Khosravi Y, Asilian-Mahabadi H, Hajizadeh E, Hassanzadeh-Rangi N, Bastani H, Khavanin A (2013). Modeling the factors affecting unsafe behavior in the construction industry from safety supervisors' perspective. J Res Health Sci.

[21] Khosravi Y, Asilian-Mahabadi H, Hajizadeh E, Hassanzadeh-Rangi N, Behzadan AH (2014). Structural modeling of safety performance in construction industry. Iran J Publ Health.

[22] Ko SM, Ji YG (2018). How we can measure the non-driving-task engagement in automated driving: comparing flow experience and workload. Appl Ergon.

[23] Mehler B, Reimer B, Coughlin J, Dusek J (2009). Impact of incremental increases in cognitive workload on physiological arousal and performance in young adult drivers. Transport Res Rec.

[24] Melo MO, da Silva LB, dos Santos Rebelo F (2017). Ergonomics aspects and workload on the operators in the electric power control and operation centers: multi-case studies in Portugal and Brazil. Iberoamerican J Ind Eng.

[25] Mohammadi A, Tavakolan M, Khosravi Y (2018). Developing safety archetypes of construction industry at project level using system dynamics. J Saf Res.

[26] Mohammadi A, Tavakolan M, Khosravi Y (2018). Factors influencing safety performance on construction projects: A review. Saf Sci.

[27] Paxion J, Galy E, Berthelon C (2014). Mental workload and driving. Front Psychol.

[28] Pickup L, Wilson JR, Sharpies S, Norris B, Clarke T, Young MS (2005). Fundamental examination of mental workload in the rail industry. Theor Issues Ergon Sci.

[29] Ryu K, Myung R (2005). Evaluation of mental workload with a combined measure based on physiological indices during a dual task of tracking and mental arithmetic. Int J Ind Ergon.

[30] Senge PM (2006). The fifth discipline: The art and practice of the learning organization.

[31] Sterman JD (2001). System dynamics modeling: tools for learning in a complex world. California Managem Rev.

[32] Teo G, Reinerman-Jones L, Matthews G, Szalma J (2015). Comparison of measures used to assess the workload of monitoring an unmanned system in a simulation mission. Procedia Manufacturing.

[33] Wascher E, Getzmann S, Karthaus M (2016). Driver state examination—Treading new paths. Accid Anal Prev.

[34] Wilms MS, Zeilstra MP, Dadashi N, Scott A, Wilson JR, Mills A (2013). Subjective mental workload of Dutch train dispatchers: Validation of IWS in a practical setting. Rail human factors: supporting reliability, safety and cost reduction.

[35] Xiao X, Wanyan X, Zhuang D (2015). Mental workload prediction based on attentional resource allocation and information processing. Biomed Mater Eng.

[36] Xie B, Salvendy G (2000). Review and reappraisal of modelling and predicting mental workload in single-and multi-task environments. Work & Stress.

[37] Yan S, Tran CC, Wei Y, Habiyaremye JL (2019). Driver’s mental workload prediction model based on physiological indices. Int J Occup Saf Ergon.

[38] Yim NH, Kim SH, Kim HW, Kwahk KY (2004). Knowledge based decision making on higher level strategic concerns: system dynamics approach. Exp Syst Applic.

[39] Young MS, Brookhuis KA, Wickens CD, Hancock PA (2015). State of science: mental workload in ergonomics. Ergonomics.

[40] Yu D, Lowndes B, Thiels C, Bingener J, Abdelrahman A, Lyons R (2016). Quantifying intraoperative workloads across the surgical team roles: room for better balance?. World J Surg.

[41] Zhao X, Hsu CY, Chang PC, Li L (2016). A genetic algorithm for the multi-objective optimization of mixed-model assembly line based on the mental workload. Engin Applic Artif Intell.

