# Supplementary material for: Human-based dynamics of mental workload in complicated systems
Source: EXCLI J. 2019 Jul 11;18:501–12. doi: 10.17179/excli2019-1372 (PMC6694705; doi:10.17179/excli2019-1372)
Supplement: Supplementary material [file EXCLI-18-501-s-001.pdf]

EXCLI Journal 2019;18:501-512 – ISSN 1611-2156
Received: April 24, 2019, accepted: July 08, 2019, published: July 11, 2019

Supplementary material to

HUMAN-BASED DYNAMICS OF MENTAL WORKLOAD IN COMPLICATED SYSTEMS

Mohammad-Javad Jafari[1], Farid Zaeri[2], Amir H. Jafari[3], Amir T. Payandeh Najafabadi[4], Narmin Hassanzadeh-Rangi*[5]

[1] Department of Occupational Health and Safety Engineering, School of Public Health and Safety, Shahid Beheshti University of Medical Sciences, Tehran, Iran. E-mail: m_jafari@sbmu.ac.ir

[2] Proteomics Research Center and Department of Biostatistics, Faculty of Paramedical Sciences, Shahid Beheshti University of Medical Sciences, Tehran, Iran. E-mail: fzayeri@gmail.com

[3] Medical Physics & Biomedical Engineering Department, School of Medicine, Tehran University of Medical Sciences, Tehran, Iran. E-mail: h_jafari@tums.ac.ir

[4] Department of Actuarial Science, Faculty of Mathematical Sciences, Shahid Beheshti University, G.C. Evin, 1983963113. E-mail: amirtpayandeh@gmail.com

[5] Department of Occupational Health and Safety Engineering, School of Public Health and Safety, Shahid Beheshti University of Medical Sciences, Tehran, Iran

* Corresponding author: Narmin Hassanzadeh-Rangi, Department of Occupational Health and Safety Engineering, School of Public Health and Safety, Shahid Beheshti University of Medical Sciences, Tehran, Iran. 7th Floor, Bldg No. 2 SBUMS, Arabi Ave, Daneshjoo Blvd, Velenjak, Tehran, Iran. Post Code: 19839-63113, Tel: +98 21 22432040, E-mail: narminhassanzadeh@sbmu.ac.ir

http://dx.doi.org/10.17179/excli2019-1372

This is an Open Access article distributed under the terms of the Creative Commons Attribution License (http://creativecommons.org/licenses/by/4.0/).

EXCLI Journal 2019;18:501-512 – ISSN 1611-2156
Received: April 24, 2019, accepted: July 08, 2019, published: July 11, 2019

Supplementary Table 1: A sample table of characteristics of included studies

Reference	Reference type	Study design	Method of data collection	Instru-ment	Field	Set-ting	Analytical method	Variables and key results	Qual-ity rat-ing
Luque-Casado et al., 2016	Biological Psychology	Experi-ment: measure develop-ment	Twenty-four males under-graduate stu-dents	Heart rate variability (HRV) and NASA-TLX	Execution condi-tion including the psychomotor vig-ilance task, a working memory task and a dura-tion discrimina-tion task	Com-puter-ized simu-lation	ANOVA and correlation	HRV varied as a func-tion of task demands. A significant decre-ment in HRV as a function of time-on-task. The NASA-TLX varied as a function of cognitive workload.	**Good**
Fallahi et al., 2016	Applied Ergonomics	Experi-ment and a cross-sectional study: oc-cupa-tional health	Physiological signals (ECG, EMG) were rec-orded and the NASA-Task Load Index (TLX) was ad-ministered for 16 operators.	NASA-TLX, ECG and EMG	Traffic density monitoring	Real	ANOVA, Bonferroni multiple comparison, Greenhouse-Geisser cor-rection	The findings indicated that increasing traffic congestion had a sig-nificant effect on HR, RMSSD, SDNN, LF/HF ratio, and EMG amplitude.	**Good**
Charbonnier et al., 2016	Expert Sys-tems with Applications	Experi-ment: measure develop-ment	15 subjects per-formed a tedi-ous but mentally demanding task on a computer during 90 min.	Karolinska Sleepiness Scale and EEG, EOG	Memory tasks	Com-puter-ized simu-lation	Signal analy-sis: time–fre-quency anal-ysis	The index based on the alpha band is well correlated with an ocu-lar index that measures external signs of mental fatigue over long periods of time.	**Good**

EXCLI Journal 2019;18:501-512 – ISSN 1611-2156
Received: April 24, 2019, accepted: July 08, 2019, published: July 11, 2019

Supplementary Table 2: The main and sub-factors (evidences) of mental workload, along with cited studies

Factor	Sub-factor (evidence)	Number of citations
Task demand and job characteristics	Task complexity	15
	Task difficulty	9
	Time constraint	7
	Speed	2
	Shift work	2
	Multitasking	1
	New technology	1
External and environmental stress	Heat stress	6
	Noise	4
	Air quality	1
	Lighting	1
	Hygienic conditions	1
Individual capabilities and characteristics	Mental and subjective capacity	196
	Autonomic sympathetic and parasympathetic response	156
	Cardiovascular capacity	140
	Mental response (brain activity)	127
	Cognitive ability and psychomotor performance	66
	Mental health, feeling and disorder	27
	Demographic characteristics	6

EXCLI Journal 2019;18:501-512 – ISSN 1611-2156
Received: April 24, 2019, accepted: July 08, 2019, published: July 11, 2019

Supplementary Table 3: Equation of some variables with inputs and initial value

**Mental Workload (Level)** = INTEG (Mental Workload Rate-Mental Workload Modification, 0)
**Mental Fatigue (Level)** = INTEG (Mental Workload-Mental Workload Modification, 0)
**Resource Supply (Level)** = INTEG (Resource Recovery Rate-Resource Consumption Rate, 100)
**Psycho-physiological Response (Auxiliary)** = WITH LOOKUP (Mental Workload*Individual Characteristics) ([(0,0)-(200,180)], (0,65), (27.5229, 93.1579), (110.092, 131.842), (195.719, 142.105), (199.388, 143.684))
**Performance Pressure (Auxiliary)** = ABS (100-Task Performance)
**Task Performance (Auxiliary)** = (Resource Supply*Productivity Ratio)
**Mental Workload Rate (Auxiliary)** = ABS (Task Demand*2*((External Stress + Performance Pressure + Time Constraint)/300))
**Mental Workload Modification (Auxiliary)** = (Mental Workload*Mental Workload Modification Index)
**Individual Characteristics (Constant)** = 1
**Resource Recovery Rate (Auxiliary)** = WITH LOOKUP (Psycho-physiological Response*Resource Recovery Index) ([(0,0)-(180,1)], (65,0), (93.0275, 0.109649), (103.486, 0.153509), (108.44, 0.179825), (113.945, 0.214912), (129.908, 0.337719), (153.578, 0.872807), (160.183, 0.929825), (172.844, 0.960526), (179.45, 0.960526))
**Resource Consumption Rate (Auxiliary)** = WITH LOOKUP (Psycho-physiological Response*Resource Consumption Index) ([(0,0)-(180,1)], (65,0), (75.9633, 0.254386), (89.1743, 0.508772), (112.844, 0.754386), (137.064, 0.903509), (162.385, 0.973684), (179.45, 0.986842))

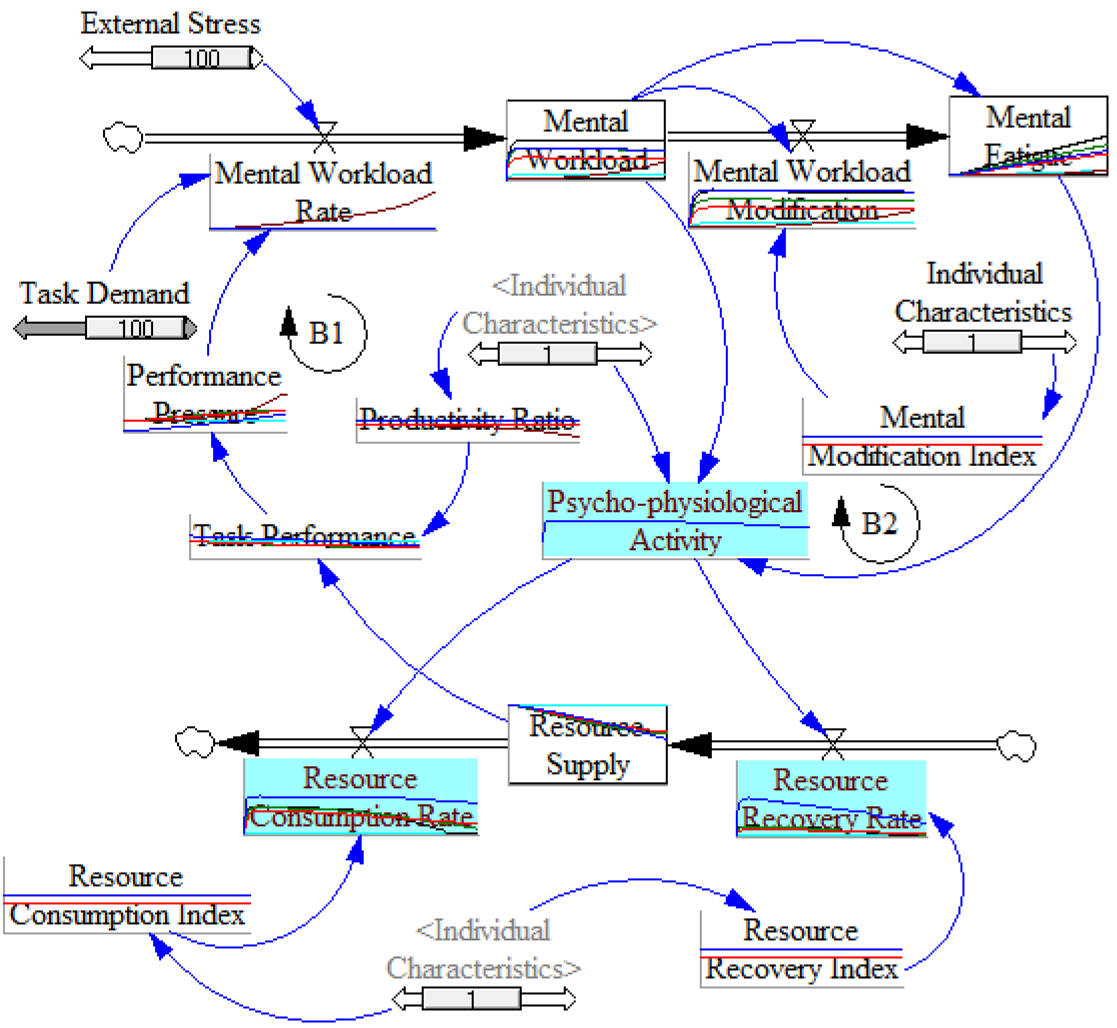

Supplementary Figure 1: Vensim software runs under different conditions on various variables in the human-based archetype of mental workload

REFERENCES

Charbonnier S, Roy RN, Bonnet S, Campagne A. EEG index for control operators' mental fatigue monitoring using interactions between brain regions. Exp Syst Applic. 2016;52:91-8.

Fallahi M, Motamedzade M, Heidarimoghadam R, Soltanian AR, Miyake S. Effects of mental workload on physiological and subjective responses during traffic density monitoring: A field study. Appl Ergon. 2016;52:95-103.

Luque-Casado A, Perales JC, Cárdenas D, Sanabria D. Heart rate variability and cognitive processing: The autonomic response to task demands. Biol Psychol. 2016;113:83-90.
